# Key Points of Endoscopic Minimally Invasive Surgery for Gynecomastia Based on Refined Anatomy: A Case Series

**DOI:** 10.1007/s00266-026-06124-3

**Published:** 2026-07-28

**Authors:** Weifang Zhang, Yueyao Sun, Mengyang An, Xin Chen, Yifang Wang, Jianping Feng, Ji Feng, Fangjian Shang, Bo Liu

**Affiliations:** Breast and Thyroid Disease Center, The First Hospital, Hebei Medical University, No. 89 Donggang Road, Yuhua District, Shijiazhuang, China

**Keywords:** Case series, Gynecomastia, Refined anatomy, Endoscopic minimally invasive surgery, Nipple-areolar complex sensation

## Abstract

**Background:**

Traditional surgical approaches for gynecomastia are associated with significant trauma and insufficient protection of anatomical structures. Currently, endoscopic glandular excision has emerged as the primary treatment modality. A refined anatomical approach for endoscopic minimally invasive surgery is proposed, with evaluation of postoperative complications and aesthetic outcomes.

**Methods:**

This study is a single-center, single-arm prospective investigation that collected and analyzed clinical data from patients who underwent endoscopic mastectomy at The First Hospital of Hebei Medical University, aiming to evaluate postoperative complications and patient recovery outcomes with an emphasis on the refined anatomical approach.

**Results:**

A total of 786 patients were included. The mean operative time was 117.8 ± 20.0 minutes, and the mean discharge time was 3.0 ± 0.7 days. Major complications included hematoma (*n* = 4, 0.5%) and nipple-areolar complex ischemia (*n* = 54, 6.9%). Among these, 52 cases of nipple-areolar complex ischemia resolved during follow-up, while 2 case progressed to necrosis (0.3%). Nipple-areolar complex sensation scores were 8.5 ± 2.0 (unilateral) and 8.0 ± 1.8 (bilateral). All procedures were completed without conversion to open surgery or significant intraoperative bleeding.

**Conclusion:**

Endoscopic glandular excision, through meticulous vascular and neural preservation, effectively restores the natural contour of the male chest while minimizing complications. This approach offers a safe and aesthetically favorable solution for gynecomastia patients.

**Level of Evidence III:**

This journal requires that authors assign a level of evidence to each article. For a full description of these Evidence-Based Medicine ratings, please refer to the Table of Contents or the online Instructions to Authors www.springer.com/00266.

**Supplementary Information:**

The online version contains supplementary material available at https://doi.org/10.1007/s00266-026-06124-3.

## Background

Current surgical options for gynecomastia include liposuction with rotary resection, periareolar open excision, liposuction with periareolar open excision, and endoscopic glandular excision [1–5]. Traditional methods often neglect critical anatomical structures, increasing postoperative risks. In contrast, endoscopic excision provides enhanced visualization, minimal trauma, concealed incisions, and thorough gland removal, establishing it as a common surgical approach for gynecomastia management in current clinical practice.

With the widespread adoption of ultra-high-definition endoscopic systems and advancements in surgeons’ technical expertise in endoscopic breast surgery, optimizing precise fascial anatomical principles to enhance operative refinement has emerged as a pressing challenge in contemporary breast surgery. Drawing on our institutional experience of over 2000 endoscopic glandular excision procedures for gynecomastia, this study elucidates the clinical significance of vascular preservation, neural protection, and layered fascial dissection in achieving surgical refinement through the clinical application of meticulous fascial anatomy in endoscopic male breast surgery.

## Materials and Methods

### Patients

The sample size was determined using the formula for single-arm study design. Based on a previous meta-analysis of surgical approaches for gynecomastia [6], the success rate with a complication-free outcome exceeded 87%. The significance level (α) was set at 0.025, and statistical power (1-β) at 80%. With these parameters, the calculated required sample size was *n* = 316.

This study included clinical records of patients who underwent endoscopic mastectomy between January 2024 and December 2024 at the Breast and Thyroid Disease Center of The First Hospital of Hebei Medical University. Data included general clinical information, operative details (duration, complications).

Inclusion criteria: preoperative diagnosis of gynecomastia via breast ultrasound or chest CT. Exclusion criteria: incomplete records or loss to follow-up.

Patient follow-up was conducted via telephonic interviews or outpatient clinic reviews. Nipple-areolar complex(NAC) sensation was self-assessed using a 0-10 numerical scale (0: complete anesthesia; 10: normal sensation). The follow-up period concluded on February 28, 2025.

All participants underwent standardized preoperative physician-patient communication, with surgical approaches collaboratively determined by both patients and surgeons. Written informed consent was obtained prior to all procedures. This case series has been reported in line with the PROCESS Guideline [7]. This study was approved by the Ethics Committee of The First Hospital of Hebei Medical University. The research adheres to the revised version of the Declaration of Helsinki.

### Surgical Technique

**Fig. 1:**
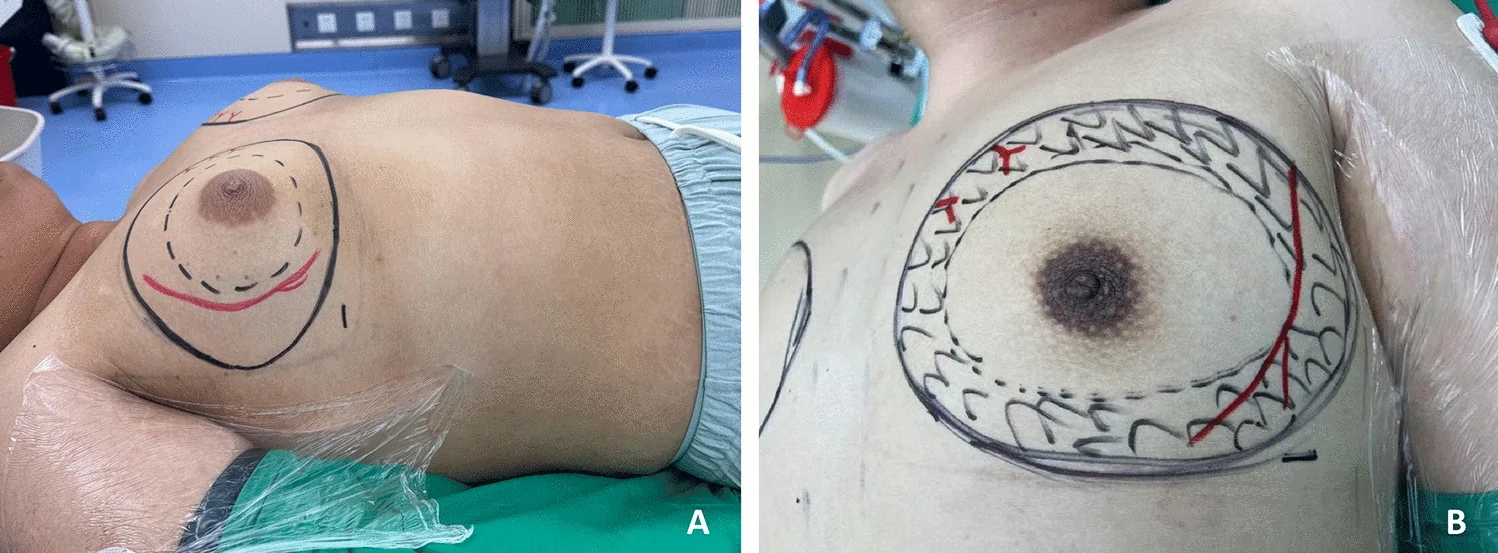
**A** Patient positioning. **B** Preoperative marking of liposuction zones and critical neurovascular structures

*Step* 1: Anesthesia and Positioning: General anesthesia with endotracheal intubation. Patients were placed supine with arms adducted and elbows flexed to expose the anterior axillary line (Fig. 1A).

*Step* 2: Preoperative Marking: Mapping was systematically performed to delineate glandular excision area and liposuction boundaries as the “dual-ring” zones, with particular focus on mapping the anatomical courses of predominant vasculature including the lateral thoracic arteries/veins and internal thoracic arteries/veins (Fig. 1B).

**Fig. 2:**
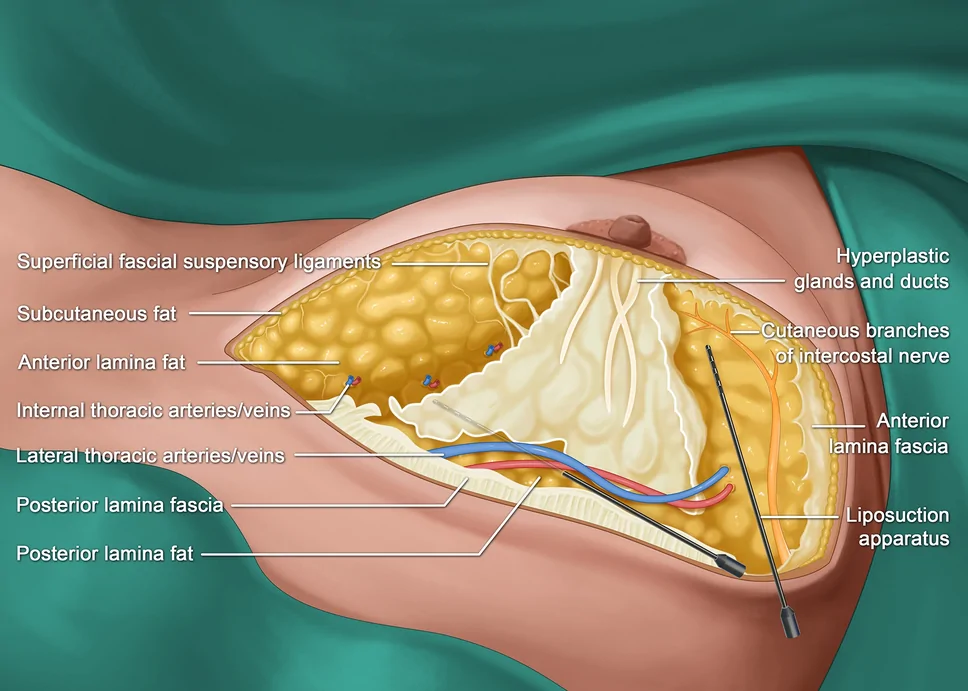
Inferior view of the neurovascular structures and fascial system in the male breast, concurrently depicting the surgical field schematic for endoscopic glandulectomy

**Fig. 3:**
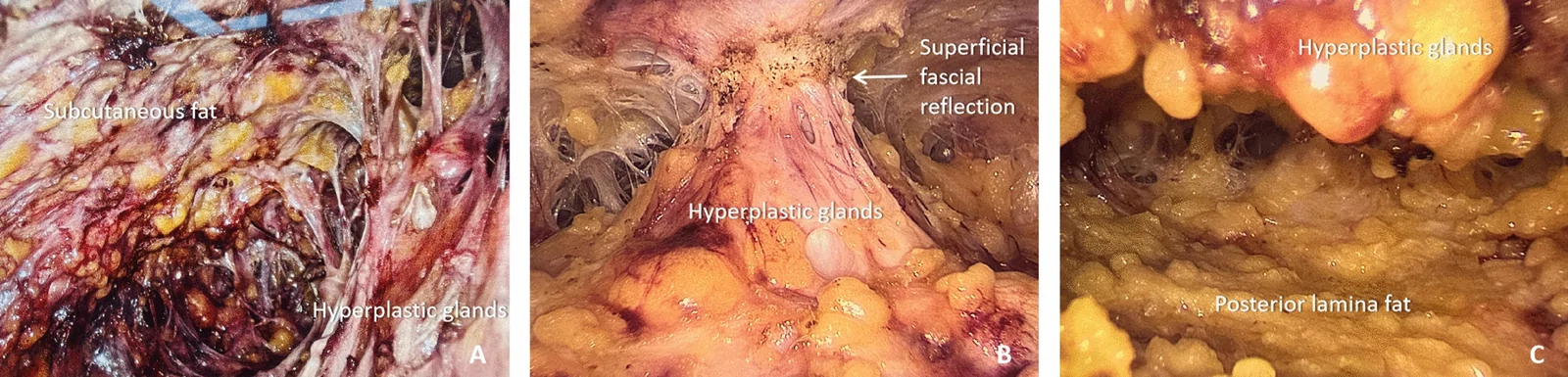
**A** Intact preservation of subcutaneous fat overlying the superficial layer of the superficial fascia. **B** Subareolar suspension of hyperplastic ducts and glands, with arrows highlighting the fascial reflection of the superficial fascia. **C** Uniform fat retention on the deep layer of the superficial fascia

*Step* 3: Surgical Planes: A 1-cm incision was made at the intersection of the inframammary fold and anterior axillary line. Lipolytic solution was evenly injected into both the anterior mammary space and posterior mammary space. Twenty minutes later, liposuction was performed using standard liposuction apparatus. During liposuction of the anterior and posterior mammary space, a “dual-plane” architecture is established. The “dual-plane” of the liposuction apparatus is demonstrated in Fig. 2. Dissection was carried out along the deep aspect of the superficial layer of the superficial fascia, within the avascular plane. The cannula was oriented with its lateral port facing the glandular surface to maintain optimal flap thickness (Fig. 3A). Aspiration of the fat adherent to the glandular surface (anterior lamina fat) revealed the capsule of the corpus mammae. During aspiration of the posterior mammary space, position the side port facing the basal surface of the gland to preserve a uniform layer of fat on the posterior lamina fascia, while aspirating the deep adipose tissue adhering to the gland. This technique aims to retain the fat pad (posterior lamina fat) overlying the posterior lamina fascia. (Fig. 3C). The liposuction extent was required to reach the periphery of the outer ring. Particular attention was paid to preserving adequate lateral flap thickness to prevent injury to the cutaneous branches of intercostal nerves.

**Fig. 4:**
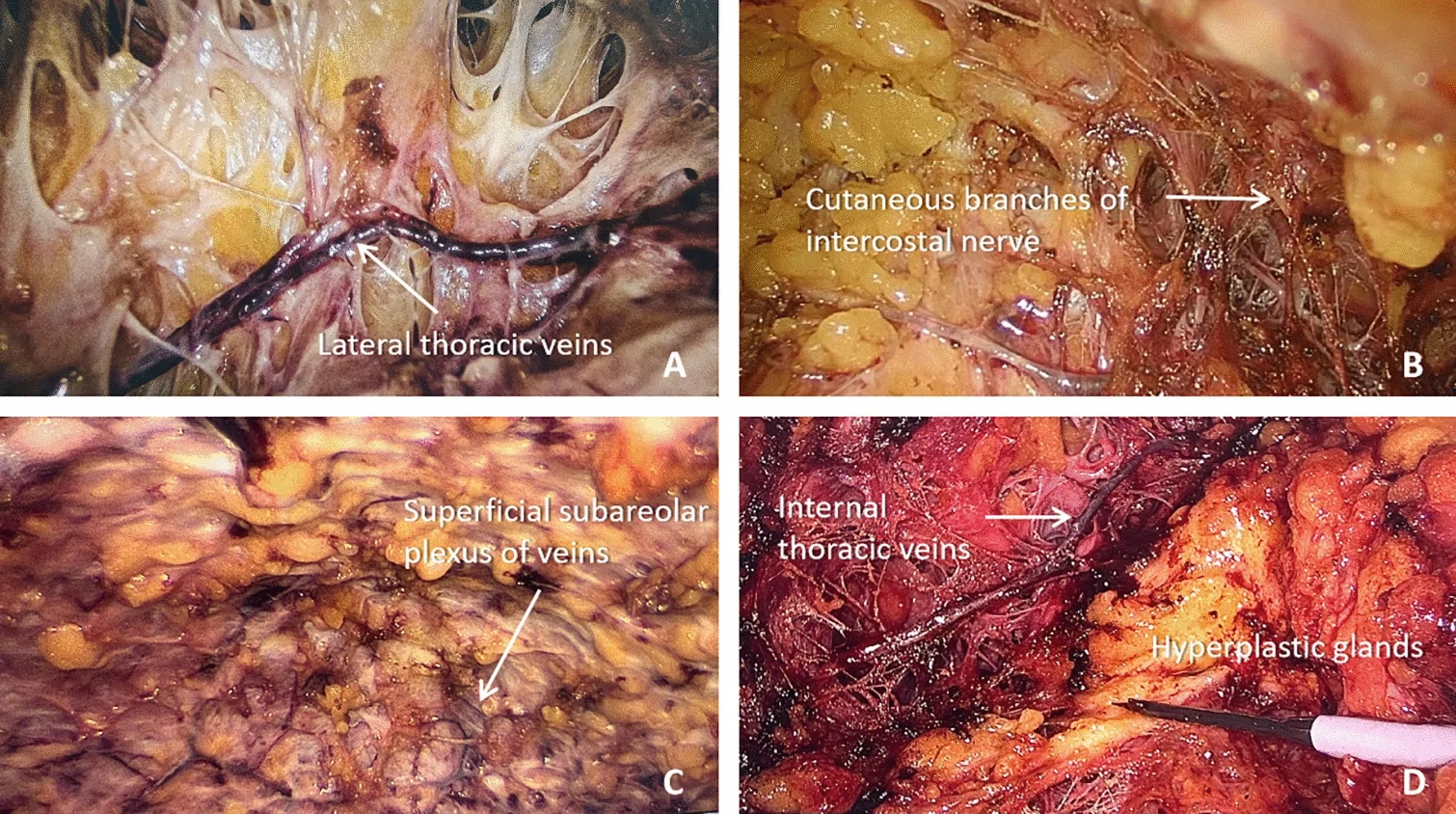
**A** Preserved lateral thoracic vein. **B** Exposed cutaneous branches of intercostal nerves. **C** Superficial subareolar plexus of veins with branches converging laterally and medially into superior venous tributaries. **D** Perforating branches of the internal thoracic vein

*Step* 4: Glandular Resection: After liposuction, a 10-mm trocar was introduced through the 1-cm inframammary fold incision to establish the operative cavity with CO₂ insufflation at 8 mmHg. A 3-mm axillary stab incision accommodated a needle-tip electrosurgical device for complete transection of superficial fascial suspensory ligaments overlying the glandular surface along the superficial aspect of the “dual plane” framework. During superficial dissection, meticulous preservation of mammary margins was prioritized to prevent injury to major vascular trunks. During the dissection of the superficial lateral layer, the constant thoracolateral vein is frequently visualized (Fig. 4A), while internal thoracic vein perforators are rarely encountered (Fig. 4D). For severely hypertrophic breasts with redundant skin, it is essential to release the horizontal ligament along the inferior mammary fold to enhance skin compliance and prevent postoperative skin folds. Particular attention should be paid to preserving the transverse neural branches, especially the cutaneous branches of intercostal nerves along the lateral glandular margin (Fig. 4B).

The ligaments along the inferior border of the gland were further resected following the deep aspect of the “dual-plane” layer. Complete excision of the glandular tissue within the inner ring was performed. During deep glandular dissection, meticulous preservation of the main trunks of the intercostal vessels perforating through the pectoralis major muscle and the lateral thoracic vessels was ensured. The schematic diagram of the surgical field is illustrated in Fig. 2.

*Step* 5: Subareolar dissection: After thorough liposuction of the subareolar adipose tissue, the reflection of the superficial fascia at the areolar region was fully exposed (Fig. 3B). The needle-tip electrosurgical device was utilized in low-power mode to transect the subareolar tissue, leaving a uniform thickness of 3 mm. Special attention was paid to preserving the subareolar plexus of veins (Fig. 4C). Following complete glandular resection, the mammary gland was extracted through a 1 cm incision at the inframammary fold. Meticulous hemostasis was achieved, and the procedure concluded with standard wound closure.

### Statistical Analysis

Data were analyzed using SPSS 25.0. Continuous variables are expressed as mean ± SD and categorical variables are described using frequencies and percentages. Continuous variables were analyzed using the two-tailed Student’s t-test, and categorical variables were analyzed using the χ2 test or Fisher’s exact test, as appropriate. A P-value < 0.05 was considered statistically significant.

## Results

A total of 786 male patients were enrolled, including 13 unilateral cases and 773 bilateral cases. The mean age was 28.0 ± 9.6 years. The mean BMI was 26.8 ± 4.0. Simon classification was as follows: Grade I (*n* = 114, 14.5%), Grade IIa (*n* = 178, 22.6%), Grade IIb (*n* = 416, 53.0%), and Grade III (*n* = 65, 8.3%).

All procedures were successfully performed with no conversion to open surgery, no massive hemorrhage during surgery and no closed drains placed. The mean operative time was 117.8 ± 20.0 minutes, and the mean discharge time was 3.0 ± 0.7 days (Table 1). Postoperative complications included hematoma (*n* = 4, 0.5%) and nipple-areolar ischemia (*n* = 54, 6.9%). Of the 54 ischemic cases, 52 resolved spontaneously during follow-up, while two progressed to partial nipple-areolar necrosis (0.3%). Sensory evaluation scores for the NAC were 8.5 ± 2.0 (unilateral) and 8.0 ± 1.8 (bilateral), respectively (Table 2). The compression garment was worn for one month after the operation, and the mean follow-up time was 96.5 ± 5.0 days.

**Table 1:** Surgical characteristics of patient cohort (*N* = 786)

Characteristic	*n* (%)
Laterality	
Bilateral	773 (98.3%)
Unilateral	13 (1.7%)
Mean age ± SD	28.0 ± 9.6
Simon	
Ia	114 (14.5%)
IIa	178 (22.6%)
IIb	416 (52.9%)
III	65 (8.3%)
Mean operative time ± SD	117.8 ± 20.0
Mean discharge time ± SD	3.0 ± 0.7

**Table 2:** Surgical outcomes of patient cohort (*N* = 786)

Characteristic	*n* (%)
NAC sensation	
Mean bilateral sensation	8.0 ± 1.8
Mean unilateral sensation	8.5 ± 2.0
Hematoma	4 (0.5%)
NAC superficial necrosis	2 (0.3%)
NAC ischemia	54 (6.9%)
Infection	0
Open surgery	0

## Discussion

Over the past few decades, various surgical approaches for gynecomastia have been explored, with substantial evidence comparing their advantages and limitations. We retrieved the literature on surgical methods for gynecomastia and their complications, as detailed in Table 3. Among them, Xu et al. compared the periareolar incision excision and axillary approach endoscopic excision. The results showed that the rate of hematoma complications in endoscopic surgery was significantly lower than that in open excision [12]. The possible reason for the analysis might be that, under the same surgical scope as laparoscopic surgery, the incision of open surgery is larger, which leads to greater damage. Moreover, laparoscopy offers a magnified view several times that of open surgery, making the exposure of key structures clearer. Sim et al. evaluated differences among microdebrider excision and liposuction technique, liposuction only, open excision only and liposuction and open excision technique. The microdebrider excision and liposuction technique approach demonstrated advantages in reducing hematoma (4.5%) and scarring (1.9%), though its main disadvantage was glandular remnant (11.5%) [1]. In contrast, laparoscopic surgery can more thoroughly remove the gland. Yang et al. reported transaxillary single-incision endoscopic mastectomy combined with liposuction, observing unilateral subcutaneous seromas in 3 out of 89 breasts (3.4%) and unilateral hematomas in 5 cases (5.6%) [5]. The reported complication rate is also higher than that of the 1-cm single-port laparoscopic technique we have presented. Chen et al. compared the clinical efficacy of subcutaneous gland resection through an areolar incision, liposuction combined with single-hole endoscopic subcutaneous gland resection, and liposuction combined with three-hole endoscopic subcutaneous gland resection. Complication rates for hemorrhage and hematoma were 1 (5%), 1 (3.6%), and 0 (0%) across the three groups, respectively, while abnormal sensation of nipple and chest skin occurred in 4 (20%), 7 (25%), and 9 (31%) cases [2]. Overall, these previous studies largely corroborate our findings, demonstrating that endoscopic glandular resection exhibits advantages in preventing complications such as hematoma, nipple-areolar sensory loss, and scarring, and has refined the surgical procedures. The following discussion addresses critical operative steps based on these anatomical refinements.

**Table 3:** Comparison of different surgical methods

Author	Year	Surgical Technique	Sample size	Follow-up (months)	Complications(%)					Hospital stay
					Hematoma	Hypoes thesia	NAC necrosis	Scar	Others	
Sim et al. [1]	2020	Microdebrider excision and liposuction technique (MELT)	156	12	4.5	–	–	1.9	Residual breast (11.5),	–
		Liposuction only (LO)	52	12	5.8	–	–	0	Residual breast (19.2)	–
		Excision only (EO)	48	12	8.3	–	–	2.1	Residual breast (12.5)	–
		Liposuction and open excision technique (LET)	48	12	8.3	–	–	2.1	Residual breast (8.3)	–
Yao et al. [3]	2019	Vacuum-assisted mastectomy	22	6.1	3.0	3.0	–	–	–	4.5d
Chen et al. [2]	2024	Areola incision + Liposuction	20	2	5.0	20.0	–	–	Subcutaneous effusion (20.0) Wound infections(10.0)	4.5d
		Liposuction + Single-port endoscopic glandectomy	28	2	3.6	25.0	–	–	Wound infections(3.6)	3.5d
		Liposuction + Triple-port endoscopic glandectomy	29	2	–	31.0	–	–	Subcutaneous effusion (3.4), Glandular residues(3.4)	4.5d
Diyarbakirlioglu et al. [8]	2019	Inferior pedicled non-vertical scar reduction technique	58	4–12	3.4	1.7	–	–	–	–
Qu et al. [9]	2020	Open surgery	56	31.5	1.8	8.9	–	–	Bruise (8.9), delayed agglutination (5.4)	5.5d
		Vacuum-assisted mastectomy using Mammotome	13	31.5	7.7	7.7	–	–	Bruise (30.8)	3.1d
		Vacuum-assisted mastectomy using Encor	14	31.5	14.3	7.1	–	–	Bruise (35.7)	3.1d
Hasan [10]	2020	Subcutaneous mastectomy using periareolar incision with lateral and medial extension needed according to Webster	100	6	31	10	9	2	Bruise (20.0), soft tissue deformity (60.0), seroma (17.0), wound dehiscence (3.0)	–
		Proposed Modified Benelli Technique	105	6	9	14	6	1	Hematoma (8.6), bruise (2.9), soft tissue deformity (19.0), seroma (11.4), wound dehiscence (4.8), areolar epidermolysis (5.7),	–
Wang et al. [11]	2019	Traditional open surgery	30	12	–	–	–	–	Edema (16.7), bruise (20.0)	7.2d
		Mammotome-assisted minimal invasive resection	30	12	–	–	–	–	Edema (46.7), bruise (53.3)	2.6d
Xu et al. [12]	2019	Periareolar open excision	27	9	7.4	–	–	–	–	–
		Endoscopic-assisted axillary approach (endoscopic axillary subcutaneous mastectomy)	25	9	4.0	–	–	–	Seroma (4.0), wound dehiscence (4.0)	–
Lee et al. [13]	2019	Subcutaneous mastectomy with liposuction + postoperative	52	48	3.8	–	–	–	Seroma (5.8)	–
		Subcutaneous mastectomy with liposuction	1621	47	2.5	–	–	–	Seroma (2.2), reoperation for skin contour correction (0.5)	–
Lee et al. [14]	2018	Subcutaneous mastectomy with liposuction	1156	30	2.6	–	–	2.2	Seroma (0.7), reoperation (0.6)	–
Ozalp et al. [15]	2017	Suction-assisted liposuction	21	17.8	14.3	14.3	4.8	–	Seroma (9.5), persistent pain (23.8)	–
Choi et al. [16]	2017	Subcutaneous mastectomy with power-assisted liposuction	71	18.6	2.8	5.6	–	2.8	Seroma (4.2), inverted nipple (2.8), contour irregularity (1.4)	8.5h
Khalil et al. [17]	2016	Liposuction pull-through technique	52	6	–	19.2	–	–	Revision (1.9)	–
Kasielska-Trojan et al. [18]	2017	Adenectomy as per Webster’s technique	50	6	6.0	8.0	–	–	–	–
Kim et al. [19]	2016	Liposuction	16	23.8	–	–	–	–	Undercorrection (6.3)	–
		Liposuction and subcutaneous mastectomy	48	33.1	–	–	–	2.1	Undercorrection (2.1), dimpling deformity (2.1)	–
Ibrahiem [20]	2016	Combined ultrasound-assisted liposuction and subcutaneous mastectomy with preservation of the NAC on top of superior pedicle NAC flap through circumareolar incision	27	48	3.7	–	3.7	7.4	Skin loss (3.7),	–
Schröder et al. [21]	2015	Liposuction	53	6	3.8	–	–	–	Relapse and reoperation (3.8)	1.2d
		Liposuction through an inframammary-fold incision and bilateral excision of the glandular tissue by a minimal periareolar approach	53	6	3.8	–	–	–	Relapse and reoperation (3.8)	1.2d
Pilanci et al. [22]	2014	Gland excision + autologous fat injection	52	16	1.9	–	–	–	Lump (3.8), depression beneath areola (1.9)	–
Fisher et al. [23]	2014	Subcutaneous mastectomy via a caudal semi areolar incision	11	34	–	–	–	9.0	Unilateral nipple retraction (9.1), recurrence (9.1)	4.5d
		Subcutaneous mastectomy via a caudal semi areolar incision with liposuction	26	34	–	–	–	–	Unilateral nipple retraction (3.8), recurrence (3.8)	4.5d
Arvind et al. [24]	2014	Liposuction	6	6	3.8	–	–	–	Seroma (3.8), superficial wound dehiscence (3.8), minor bleeding (5.7)	1.6d
		Excision (semi-circular incision along the inferior margin of the	37	6						
		Liposuction with excision (semi-circular incision along the inferior margin of the NAC	10	6						
Kasielska et al. [25]	2013	Subcutaneous mastectomy with circumareolar approach	94	6	3.2	2.1	–	–	Seroma (1.1)	–
		Subcutaneous mastectomy with skin excision lateral wedge	9	6	22.2	55.6	11.1	–	Wound infection (11.1),	–
		Inverted T reduction mastopexy	6	6	33.3	50.0	–	–	Seroma (50.0)	–
		Breast amputation with inframammary fold approach	4	6	25.0	25.0	–	–	–	–
Blugerman et al. [26]	2012	Open RFAL with minimal access glandular technique, trans-mammillary glandular resection	12	12	16.7	–	–	–	Seroma (8.3)	–
		Closed radiofrequency-assisted liposuction RFAL	47	12	–	–	–	–	–	–
Li et al. [27]	2012	Mastectomy using concentric circumareolar approach with nipple reposition	41	7.5	9.8	–	–	–	Seroma (2.4)	–
		Subcutaneous mastectomy with pull-through technique + ultrasound-assisted liposuction								
		Ultrasound-assisted liposuction								
Cigna et al. [28]	2010	Power-assisted liposuction and mastectomy through a semi-circular subareolar incision	37	12	2.7	–	–	–	–	–
El-Sabbagh et al. [29]	2016	Liposuction (suction-assisted) and surgical excision through a periareolar incision	14	24	–	–	14.3	–	Seroma (21.4), slight nipple retraction (21.4), superficial infection (7.1)	1.0d
Cao et al. [30]	2013	Endoscopic subcutaneous mastectomy via three small skin incisions made on the mid-axillary line	58	36	–	–	5.2	–	–	3.0-4.0d
Taheri et al. [31]	2016	Periareolar incision with liposuction	27	6	–	25.9	14.8	3.7	Chest wall asymmetry (3.7)	–
Pfeiler et al. [32]	2021	Subcutaneous mastectomy	60	69.6	5.0	–	–	–	Surgical site infection (1.7), seroma (1.7)	3.6d
		Subcutaneous mastectomy + ultrasound-assisted liposuction	45	89.2	4.4	–	–	–	Surgical site infection (2.2)	4.2d
Akhter et al. [33]	2019	Liposuction combined with open disc excision, via a periareolar incision in the lower half of the nipple-areolar complex	30	6	6.7	–	–	–	Contour irregularities (6.7), asymmetry (26.7)	–
		Liposuction coupled with disc excision via suction-assisted arthroscopic shaver	30	6	10.0	–	–	–	Contour irregularities (6.7), asymmetry (33.3), reoperation (6.7)	–

### Breast Fascia and Anteroposterior Compartments

We observed that a fascial system analogous to the thyroid capsule can also be divided into two layers in the male breast: the superficial fascia surrounding the mammary gland and adipose tissue externally and the capsule of the corpus mammae closely adhering to the glandular parenchyma. The superficial fascia comprises superficial and deep layers, analogous to Camper’s fascia and Scarpa’s fascia of the abdominal wall [34, 35]. The mammary gland suspended between the two layers of the superficial fascia, so the two layers of superficial fascia is also called the anterior lamina fascia and posterior lamina fascia. The anterior lamina fascia divides subcutaneous fat into a surface sheet that adheres to the dermis and a deep sheet with a trabecular pattern directed toward the glandular parenchyma [35, 36]. This demonstrates the superficial aspect of the “dual-plane” framework during liposuction.

**Fig. 5:**
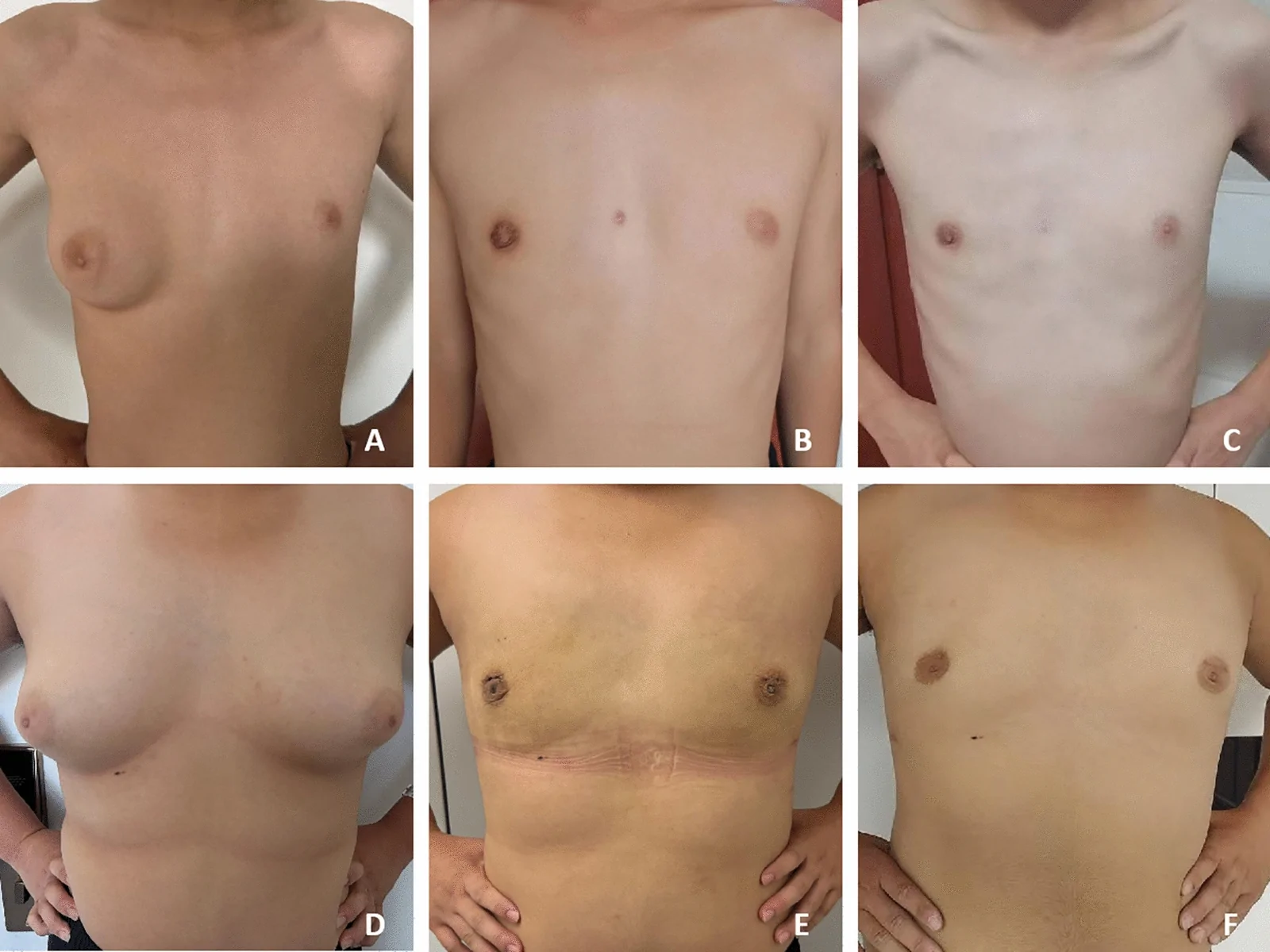
**A** Preoperative Simon grade III gynecomastia. **B** Natural breast contour demonstrated at 3-month postoperative follow-up. **C** Natural breast contour demonstrated at 1-year postoperative follow-up. **D** Preoperative right-sided Simon grade IIb gynecomastia. **E** Bilateral symmetry achieved at 3-month postoperative follow-up. **F** Bilateral symmetry achieved at 1-year postoperative follow-up

The posterior lamina fascia lies behind the gland. Between the posterior lamina fascia and the pectoral fascia, a loosely organized space termed the retromammary space allows natural breast mobility on the chest wall [36]. During surgery, liposuction and tissue dissection along the deep aspect of the “dual-plane” framework preserves a uniform layer of fat over the deep layer of Scarpa’s fascia, which we consider optimal for achieving natural aesthetic outcomes in clinical practice. Fig. 5 illustrates postoperative recovery at three month and 1 year in two patients with preoperative bilateral Simon Grade III hypertrophy and right-sided IIb hypertrophy, respectively. Postoperative breast skin contracted effectively, resulting in a natural and aesthetically pleasing contour.

### NAC Blood Supply

A male-focused study evaluated 142 thoracic computed tomographic angiography and 284 breast vasculatures. The study revealed that 69.0% of breasts were supplied by a single artery, 6.0% were fed by vascular anastomosis formed by dual arteries. Notably, 25.0% of breasts lacked identifiable dominant arterial patterns. The lateral thoracic artery (LTA) was the most frequent single dominant artery of origin (41.9%) , followed by perforating branches of the internal thoracic artery (ITA;22.2%); 4.2% of male breasts were supplied mostly by the anterior intercostal artery (AICA). The most common anastomoses were formed by the ITA and LTA [37]. Preoperative marking of key vasculature and meticulous intraoperative preservation of critical arteries/veins were implemented to minimize perfusion compromise.

In female NAC vascular studies, ITA perforators predominated as the primary supply (81.8%), followed by LTA contributions (23.5%) and AIA perforators (15.9%), with minimal involvement from the superior thoracic artery (0.8%) [38]. Two additional studies corroborated ITA as the most prevalent dominant arterial source in female NACs, succeeded by LTA [39, 40]. The areas where these vessels emerge through the thoracic wall (hypervascular zones/fixed skin areas) are constant and include the parasternal border, submammary fold, and the lateral thoracic wall along the lower lateral border of the pectoralis minor muscle [40]. Postoperative hematomas occurred in only 0.5% of cases (exclusively unilateral), underscoring the importance of avoiding mammary margin dissection during liposuction and glandular resection to preserve vascular integrity. This protocol has demonstrated significant reduction in postoperative hematoma rates compared to prior methodologies [1–3].

The venous drainage of the breast is composed of an extensive vascular network. The nipple-areolar complex (NAC) is drained by the superficial subareolar plexus of veins, which converges into the medial and lateral mammary veins. The lateral, superolateral, and inferolateral veins ultimately drain into the subclavian vein, while two medial dominant veins drain the breast tissue to the internal mammary veins. The medial vessels coursing superficially, whereas laterally the veins have a deeper course [41]. However, our analysis of 52 cases with postoperative NAC ischemia revealed intraoperative absence of the superficial subareolar plexus of veins in 36 patients (67.9%), potentially attributable to iatrogenic disruption during surgery or congenital absence of this vascular network. These findings suggest that deficiencies in the subareolar venous plexus may contribute to ischemic complications. The anatomical consistency of this venous plexus in male breasts warrants further investigation. Additionally, excessive intraoperative coagulation of venous channels might predispose to postoperative venous congestion. All affected cases demonstrated restored perfusion by the 3-month follow-up interval. The subcutaneous arteriovenous network plays a critical role in maintaining cutaneous and NAC perfusion. Preservation of this vascular network during mastectomy is imperative. Current understanding of venous anatomy indicates that injury to the superficial venous system may precipitate NAC necrosis [42].

Two cases in the cohort developed NAC necrosis with tissue sloughing at 1-month follow-up. Intraoperatively, neither significant arterial branch hemorrhage nor a discernible subareolar venous plexus was identified in this patient. The necrosis may be attributed to iatrogenic disruption of the subareolar venous plexus during liposuction (resulting in intraoperative venous stasis and postoperative congestion), compounded by ischemic compression from an excessively tight postoperative compression garment. NAC perfusion in nipple-sparing mastectomy (NSM) primarily relies on superficial vascular networks following complete glandular resection, with dominant contributions from perforating branches of the internal thoracic artery (ITA) and external thoracic artery. However, postoperative NAC necrosis cannot be entirely prevented. Critical vascular networks reside 3–5 mm beneath the areolar dermis, necessitating meticulous intraoperative preservation. Yang et al. reported no incidence of NAC necrosis in their study of 110 NSM patients when the preserved subareolar flap thickness was maintained at 3–5 mm [43]. Komiya et al. demonstrated that the anterior lamina fascia extends subareolarly, exhibiting significant thickening and reflection at an average distance of 1 cm from the nipple. Microanatomical analysis revealed that this fascial layer comprises collagenous elastic fibers interspersed with smooth muscle cells, forming a thickened reflection that encircles the lactiferous ducts, neurovascular bundles, and associated stromal structures [44]. Incision through the superficial fascial reflection at 3 mm subareolar, avoiding excessive residual tissue behind NAC to prevent postoperative “checkerboard-like” glandular tissue retention in the areola. Excessively thin residual tissue may damage the dermal blood supply of the NAC or cause postoperative nipple inversion.

### Neuroanatomy of the NAC

Cutaneous innervation of the female breast derives from anterior and lateral cutaneous branches of the second to sixth intercostal nerves (ICNs) [45]. NAC sensation is predominantly mediated by the lateral cutaneous branch of the fourth ICN, supplemented by anterior branches of the third to fifth ICNs and lateral branches of the third and fifth ICNs [46]. Substantial discrepancies exist across studies regarding the topographic course of neural pathways through breast parenchyma [45, 47–49]. It is generally recognized in that the fourth ICN’s lateral branch bifurcating into superficial and deep divisions. The deep division courses within the pectoral fascia along the inferior breast border for 3–4 cm before executing a U-shaped retrograde trajectory through inferolateral breast tissue toward the NAC, corresponding to the 4 o’clock (left) and 8 o’clock (right) positions on the breast clockface [34, 50]. Avoiding the inferolateral quadrant during liposuction and preserving adequate lateral flap thickness are critical for maintaining neural integrity within adipose tissue. Suspicious neural structures in lateral breast regions warrant meticulous preservation to optimize NAC sensory outcomes. Retaining lateral breast flaps additionally ensures natural axillary-anterior chest fat continuity, enhancing aesthetic contour. According to our follow-up results, with careful attention to nerve preservation, none of the patients experienced significant sensory deficits. Notably, some patients reported numbness during hospitalization, which showed improvement at follow-up assessments after more than 3 months of recovery. This indicates that nerve recovery requires a certain period of time, consistent with conclusions from previous studies [51].

### Limitations

This study propose the refined anatomical concept in gynecomastia surgery at a relatively early stage, providing procedural steps and technical considerations. Preliminary results suggest benefits in postoperative sensory preservation, recovery outcomes, and patient satisfaction, which warrant further validation in expanded cohorts. This study has several limitations. First, its single-center design may compromise the generalizability of the conclusions. Furthermore, as a preliminary report emphasizing surgical anatomical principles, we did not analyze factors affecting NAC sensation; current assessments rely solely on subjective patient-reported evaluations without objective measurements, a gap to be addressed through a dedicated prospective study in forthcoming research. Additionally, long-term follow-up data remain unavailable at this stage, with plans for their inclusion in subsequent investigations.

## Conclusion

Endoscopic male breast surgery should not only ensure resection of the gland but also aim to reduce postoperative complications and enhance patient satisfaction. Mastering the refined anatomy of the breast fascial layers to establish the "dual-plane" architecture prevents neurovascular injury, thereby enhancing clinical outcomes for patients with gynecomastia.

## Supplementary Information

Below is the link to the electronic supplementary material.

Supplementary file1 (MP4 200994 kb)

## Declarations

### Provenance and Peer Review

Not commissioned, externally peer-reviewed
